# Self-reported racial discrimination, response to unfair treatment, and coronary calcification in asymptomatic adults - the North Texas Healthy Heart study

**DOI:** 10.1186/1471-2458-10-285

**Published:** 2010-05-27

**Authors:** Roberto Cardarelli, Kathryn M Cardarelli, Kimberly G Fulda, Anna Espinoza, Clifton Cage, Jamboor Vishwanatha, Richard Young, Darryl N Steele, Joan Carroll

**Affiliations:** 1 Department of Family Medicine, Primary Care Research Institute, Texas College of Osteopathic Medicine, University of North Texas Health Science Center, 3500 Camp Bowie Blvd, Fort Worth, TX, 76107, USA; 2 Department of Epidemiology, School of Public Health, University of North Texas Health Science Center, 3500 Camp Bowie Blvd, Fort Worth, TX, 76107, USA; 3 Department of Integrative Physiology, Graduate School of Biomedical Sciences, University of North Texas Health Science Center, 3500 Camp Bowie Blvd, Fort Worth, TX, 76107, USA; 4 Department of Family Medicine, John Peter Smith Health Network, 1500 South Main St, Fort Worth, TX, 76104, USA; 5 Family Medicine Private Practice, HEB Family Care Clinic, Euless, TX, 76039, USA

## Abstract

**Background:**

Accruing evidence supports the hypothesis that psychosocial factors are related to cardiovascular disease. However, a limited number of studies have investigated the pathophysiologic pathways through which these associations occur. The purpose of this study was to assess whether experiences of self-reported racial discrimination and reactions to unfair treatment were associated with coronary artery calcification (CAC), an indicator of subclinical coronary heart disease (CHD).

**Methods:**

This cross-sectional study recruited 571 subjects (45 years and older) who were asymptomatic of CHD from Fort Worth, Texas from 2006 to 2008. Subjects completed a questionnaire, a multi-slice computed tomography scan to assess for CAC presence (measured as Agatston score >0), and serum chemistries. Logistic regression was used to estimate odds ratios (ORs) and 95% confidence intervals (CIs) for the association between self-reported discrimination and CAC. Results were stratified by response to unfair treatment as it was found to significantly modify the relationship between discrimination and CAC.

**Results:**

Among those who passively responded to unfair treatment, the odds of having CAC present were approximately 3 times higher for those experiencing discrimination (OR, 2.95; 95% CI, 1.19-7.32) after adjusting for age, gender, race/ethnicity, education, body mass index, hyperlipidemia, smoking status, hypertension, diabetes, and first degree relative with heart disease.

**Conclusions:**

This is the first multi-racial/ethnic study to find racial discrimination associated with CAC, which differs based on how one responds to unfair treatment.

## Background

Cardiovascular disease (CVD) remains the leading cause of death among adults and affects over 70 million people in the United States[1]. Despite marked declines in overall prevalence of CVD, racial and ethnic disparities in CVD prevalence exist[1-3]. Increasing evidence suggests that psychosocial factors may play a role in the development of CVD [4], although this is still debated[5]. Psychosocial factors including stress, depression, anger, anxiety, and lack of social support have been linked to CVD[4,6].

Racial discrimination is gaining attention as an independent factor for CVD. Conceptually defined as a source of acute and lifelong chronic stress [7-10], discrimination may contribute to CVD, indirectly by negatively impacting mental health [11,12], inducing unhealthy behavior [13], or more directly [14], by inducing inflammation and platelet aggregation, which is an underlying pathophysiologic mechanism of atherosclerosis[15]. Internalizing unfair treatment rather than talking to others about the experience may further reinforce such stress[9,16,17]. The relationship between racial discrimination and hypertension has been the focus of prior investigations, for which the results have been equivocal[18-20]. However, investigations of racial discrimination and subclinical CVD are limited[21,22].

Recent advances in computed tomography (CT) scanning have allowed for the measurement of coronary artery calcification (CAC), which may assist in detecting subclinical CVD by assessing the extent of atherosclerosis[23]. CAC is a noninvasive measure of subclinical atherosclerotic plaque calcification of the coronary arteries [23] and has been shown to independently predict coronary heart disease [6,24] and coronary events in all racial/ethnic groups[25,26]. The presence of CAC has also demonstrated variable ability to detect clinically apparent coronary artery disease[27].

A study of 181 African-American women revealed that higher levels of CAC were associated with chronic exposure to discrimination[28]. Investigators reported that for every unit increase in experiencing discrimination, there was a 2.8 fold increase in the odds of having coronary calcification. However, the study sample was limited to middle-aged women, and the measure of discrimination was not specific to racial discrimination. Available literature indicates that this study provided one of the few previous examinations of discrimination as an independent factor for CAC. Our study examined the relationship between self-reported racial discrimination, reaction to unfair treatment and CAC in a population of 571 asymptomatic adults. We hypothesized that discrimination would be associated with higher prevalence of coronary calcification and that individuals who internalized experiences of unfair treatment (i.e., passive response) would also have a higher prevalence of CAC compared to those who talked to others about such experiences (i.e, active response). To our knowledge, this represents the first study to examine these associations in a population of male and female individuals of multiple racial/ethnic groups.

## Methods

### Study population

**Figure 1:**
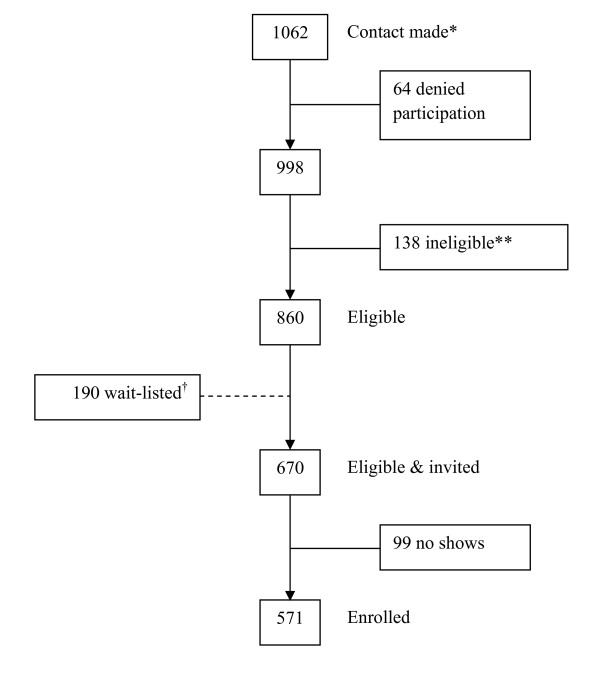
**Recruitment flow diagram**. *Recruited or participants called the research office. **Based on inclusion/exclusion criteria. †Individuals were wait-listed if their respective race/ethnicity blocks were full at the time contact was made.

The North Texas Healthy Heart (NTHH) study is a cross-sectional study involving a convenience sample of 571 non-Hispanic whites, non-Hispanic African Americans, and Hispanics/Latinos recruited from 12 participating sites of the North Texas Primary Care Practice-Based Research Network (NorTex) from April 2006 to May 2008. NorTex is a collaborative network of primary care clinics serving low-income, under-represented populations of the Dallas/Fort Worth, Texas metropolitan area. The 12 family medicine/internal medicine clinic sites that participated in the NTHH study included 4 academic community-based clinics, 3 community health centers, 4 solo-practitioner private practices, and 1 federally-qualified health center. Participants were eligible for the study if they were over the age of 44, self-identified as non-Hispanic white, non-Hispanic African American, or Hispanic/Latino, and had no history of self-reported cardiovascular disease (coronary artery disease, peripheral arterial disease, history of myocardial infarction or stroke, or congestive heart failure), renal failure, or liver failure. All participants were screened for eligibility either on-site or via phone from a centralized NorTex research office located within the University of North Texas Health Science Center, Department of Family Medicine. Initial contact was made with 1,062 individuals, with 860 meeting eligibility criteria. Of those who were eligible, 670 were invited to participant and 571 agreed to participate and the remaining were wait-listed, representing an 85% recruitment rate (Figure 1). All study procedures were approved by the University of North Texas Health Science Center and JPS Health Network Institutional Review Boards.

### Study procedures

All consented participants underwent a 1-hour face-to-face interview in a single private temperature-controlled room. Women, except for those with a history of hysterectomy, underwent a urine pregnancy test, as pregnancy is a contraindication to computed tomography scanning. There were no positive urine pregnancy tests among the study participants. Participants then completed weight, height, waist/hip circumferences, and blood pressure measures (millimeters of mercury [mmHg]). Height was measured to the nearest 0.25 inch, and weight was measured to the nearest 0.25 lb using a standard balance scale. Height and weight measurements were used to calculate a body-mass index for each subject using the Quetelet's equation (kg/m^2^)[29]. Automated Welch Allyn^© ^sphygmomanometers were used to measure heart rate and systolic and diastolic blood pressures in each arm using a size-appropriate cuff. The measures were taken after the participant was seated quietly for 5-minutes with both feet flat on the floor and the back comfortably supported. An average heart rate and systolic and diastolic blood pressure was calculated for each subject based on two separate measures.

### Demographic and health behavior measures

The NTHH study utilized standardized questions from the Behavioral Risk Factor Surveillance System to collect a selected number of demographic and health behavior information. Age was registered as a continuous variable (years). Race/ethnicity was self-reported and categorized as non-Hispanic white, non-Hispanic African American, Hispanic, and other. Education was measured by the question, "What is the highest grade or year of school that you completed?" Responses were then categorized as "less than high school", "high school graduate/GED", or "some college or greater". Smoking status was assessed by asking, "Have you smoked at least 100 cigarettes in your lifetime?" Subjects were categorized as smokers if they responded "Yes".

### Physiologic and clinical measures

The presence of coronary calcium was measured using a 16-slice MSCT scan and characterized as a dichotomous variable, consistent with previous studies [6,28,30]: presence of calcification (Agatston score (31) of greater than zero) or no calcification (Agatston score of zero). The MSCT took images every 3 mm from the carina to the base of the heart, and double inspiration was used to minimize breathing motion artifact on images. The participant had an electrocardiogram machine attached to correlate the heart rate with the images for the Vitrea software to calculate quantitative calcium scores. The total time in the scanner was 10-15 minutes, which was open with no contrast was given. Coronary artery calcification (CAC) quantification was reviewed and interpreted by a radiologist from Radiology Associates at the Center for Diagnostic Imaging at the University of North Texas Health Science Center who was blinded to participant characteristics.

Clinical factors included history of a first degree relative with heart disease, depression symptomatology, hypertension, diabetes, and lipid status. Fasting (8 hour) blood was collected for serum chemistries and analyzed using a commercial laboratory. History of a first degree relative with heart disease was categorized as yes or no. The Center for Epidemiologic Studies Depression Scale (CES-D) was used to measure depressive symptomatology (Cronbach α.85-.90)[32]. The CES-D measures current level of depressive symptomatology with emphasis on the affective component, depressed mood. The CES-D score was dichotomized into high depressive symptomatology (scores ≥ 17) and low depressive symptomatology (score < 17) based on previous research. Hypertension was considered present if the blood pressure was greater than or equal to 140/90 mm Hg for systolic or diastolic pressure, the subject reported being diagnosed with hypertension, or the subject was taking antihypertensive medications. Diabetes was considered present if the fasting glucose level was greater than or equal to 126 mg/dL, the subject reported being previously diagnosed with diabetes, or the subject was taking any diabetic medication. Hyperlipidemia was considered to be present if the participant had a LDL≥160 mg/dL, the participant reported being previously diagnosed with high cholesterol, or the participant was taking a lipid lowering medication.

### Self-reported racial discrimination and response to unfair treatment

Self-reported racial discrimination (hereafter referred to as discrimination) and response to unfair treatment were measured using the Experience of Discrimination (EOD) instrument, which was validated in English and Spanish among a population of low-wage African American, Latino, and white workers (Cronbach's alpha ≥ 0.74, r = 0.79)[16,21,33]. The EOD asks about ever experiencing discrimination, being prevented from doing something, being hassled or made to feel inferior because of race, ethnicity, or color in each of the following nine domains: at school; getting a job; at work; getting housing; getting medical care; getting service in a store or restaurant; getting credit, bank loans or a mortgage; on the street or in a public setting; or from the police or in the courts. The instrument was available to participants in English or Spanish. Discrimination was modeled as a dichotomous variable (no discrimination/any discrimination). Response to unfair treatment was measured by asking respondents, "If you have been treated unfairly, do you usually (1) talk to other people about it or (2) keep it to yourself?" Based on the instrument's guidelines, participants were categorized as passively responsive in which they internalized their action, or actively responsive in which they talked to others or did something about their experience.

### Statistical analyses

All statistical analyses were performed using SPSS version 15.0[34]. Descriptive statistics are provided for all variables. Counts and frequencies are provided for categorical data, and means and standard deviations are provided for continuous variables. Independent sample t tests and chi-square analyses were performed to test for differences in independent variables between participants with a CAC score of zero and those with a CAC score greater than zero (Table 1). Logistic regression was performed, and unadjusted and adjusted odds ratios and 95% confidence intervals were calculated (Tables 2, 3, and 4). Statistical significance was assessed at the alpha = 0.05 level.

**Table 1:** Characteristics of North Texas Healthy Heart study participants by presence of coronary artery calcification-- Fort Worth, Texas, 2006-8 (N = 510)

Variable	Any calcification, n (%)	No calcification, n (%)	p-value*
Age, mean (SD)	58.4 (8.6)	53.4 (6.9)	< .001

Body mass index, mean (SD)	32.0 (7.0)	30.4 (6.0)	0.01

Gender			< .001
Female	90 (28.4)	227 (71.6)	
Men	96 (49.7)	97 (50.3)	

Race/ethnicity			< .001
Non-Hispanic white	71 (50.0)	71 (50.0)	
Non-Hispanic African American	59 (35.3)	108 (64.7)	
Hispanic	52 (26.9)	141 (73.1)	

Education			0.39
Less than High school	34 (30.9)	76 (69.1)	
High school graduate/GED	39 (37.5)	65 (62.5)	
Some college or higher	113 (38.2)	183 (61.8)	

Smoked ≥ 100 cigarettes in one's life			0.002
Yes	95 (44.4)	119 (55.6)	
No	89 (30.8)	200 (69.2)	

Diabetes Mellitus status			0.005
Yes	46 (48.9)	48 (51.1)	
No	139 (33.7)	274 (66.3)	

Hypertension status			0.004
Yes	111 (42.7)	149 (57.3)	
No	73 (30.2)	169 (69.8)	

Hyperlipidemia status			< 0.001
Yes	119 (44.6)	148 (55.4)	
No	67 (27.8)	174 (72.2)	

First degree relative with history of coronary heart disease			0.01
Yes	106 (41.9)	147 (58.1)	
No	76 (31.3)	167 (68.7)	

Depressive symptomatology			
Low (CES-D score < 17)	135 (36.3)	237 (63.7)	
High (CES-D score ≥ 17)	50 (36.5)	87 (63.5)	

Response to unfair treatment			0.96
Active	123 (37.0)	209 (63.0)	
Passive	63 (36.8)	108 (63.2)	

Experienced racial discrimination			
Yes	125 (35.5)	227 (64.5)	
No	61 (38.6)	97 (61.4)	

* P value corresponds to a two sided p value for t-tests for continuous variables and to chi square analyses for categorical variables.

**Table 2:** Simple logistic regression models for coronary artery calcification -- Fort Worth, Texas, 2006-8 (N = 510)

	Any calcification	
**Variable**	OR*	95% CI*

Age	1.09	1.06-1.11

Body mass index	1.04	1.01-1.07

Gender		
Female	...	...
Men	2.5	1.72-3.63

Race/ethnicity		
Non-Hispanic white	...	...
Non-Hispanic African American	0.55	0.35-0.86
Hispanic	0.37	0.23-0.58

Education		
Some college or higher	...	...
Less than High school	0.97	0.61-1.54
High school graduate/GED	0.72	0.45-1.16

Smoked ≥ 100 cigarettes in one's life		
No	...	...
Yes	1.79	1.24-2.59

Diabetes Mellitus status		
No	...	...
Yes	1.89	1.20-2.97

Hypertension status		
No	...	...
Yes	1.73	1.19-2.49

Hyperlipidemia status		
No	...	...
Yes	2.09	1.44-3.03

First degree relative with history of coronary heart disease		
No	...	...
Yes	1.58	1.10-2.29

Depressive symptomatology		
Low (CES-D score < 17)	...	...
High (CES-D score ≥ 17)	1.01	0.67-1.52

Response to unfair treatment		
Active	...	...
Passive	0.99	0.68-1.45

Experienced racial discrimination		
No	...	...
Yes	0.88	0.59-1.29

* OR = crude or unadjusted odds ratio, CI = confidence interval

**Table 3:** Simple logistic regression model for the relationship between perceived racial discrimination and coronary artery calcification stratified by response to unfair racial treatment-- Fort Worth, Texas, 2006-8 (N = 510)

	Active response to unfair treatment		Passive response to unfair treatment	
**Variable**	OR*	95% CI*	OR*	95% CI*

Experienced racial discrimination				
No	...	...	...	...
Yes	0.69	0.42-1.15	1.25	0.66-2.36

OR = crude or unadjusted odds ratio, CI = confidence interval

**Table 4:** Multiple logistic regression model for the relationship between perceived racial discrimination and coronary artery calcification stratified by response to unfair racial treatment-- Fort Worth, Texas, 2006-8 (N = 510)

	Active response to unfair treatment		Passive response to unfair treatment	
**Variable**	OR*	95% CI*	**OR**	**95% CI**

Age	1.09	1.05-1.13	1.1	1.04-1.16

Body mass index	1.03	0.99-1.08	1.08	1.01-1.15

Gender				
Female	...	...	...	...
Men	3.4	1.96-5.88	2.32	1.01-5.35

Race/ethnicity				
Non-Hispanic white	...	...	...	...
Non-Hispanic African American	0.64	0.31-1.32	0.26	0.08-0.84
Hispanic	0.4	0.18-0.88	0.49	0.15-1.55

Education				
Some college or higher	...	...	...	...
Less than High school	1.1	0.46-2.62	0.33	0.10-1.06
High school graduate/GED	1.23	0.62-2.44	0.66	0.24-1.83

Smoked ≥ 100 cigarettes in one's life				
No	...	...	...	...
Yes	1.27	0.74-2.19	2.56	1.05-6.40

Diabetes Mellitus status				
No	...	...	...	...
Yes	1.91	0.94-3.89	1.35	0.46-3.99

Hypertension status				
No	...	...	...	...
Yes	1.15	0.64-2.04	1.13	0.48-2.68

Hyperlipidemia status				
No	...	...	...	...
Yes	1.64	0.95-2.83	2.08	0.86-4.99

First degree relative with history of coronary heart disease				
No	...	...	...	...
Yes	1.08	0.63-1.85	0.95	0.40-2.23

Experienced racial discrimination				
No	...	...	...	...
Yes	0.8	0.41-1.54	2.95	1.19-7.32

* OR = adjusted odds ratio, CI = confidence interval

The multiple logistic regression model assessed potential interaction for discrimination*response to unfair treatment and discrimination*race/ethnicity in the final model. Multicollinearity was assessed using Tolerance and Variation Inflation Factor (VIF) with all variables in the final model. No collinear relationships were identified. In addition, a sensitivity analysis was performed to assess whether depression symptomatology (i.e, CES-D) should be included in the final adjusted model. This was examined due to a lack of literature supporting the inclusion of depression symptomatology and the lack of significance in the unadjusted association between depression and CAC. A full regression model was composed with depression, and another model was composed without depression. The change in the -2 Log Likelihood was used to assess change in the fit of the model. Including depression symptomatology in the model decreased the -2 Log Likelihood. Missing data were imputed for discrimination and CES-D using the individual mean imputation method, which imputes a value based on how a subject responds to other questions. This method was chosen because of its simplicity and accuracy[35]. Missing data were not imputed for CAC scores (10.7% missing), unfair treatment (1.6% missing), gender (0.4% missing), education (0.4% missing), smoking (1.8% missing), diabetes (0.9%), hypertension (2.1%), hyperlipidemia (1.2% missing), 1st degree family member with a history of heart disease (3.0% missing), BMI (0.9% missing), and age (0.5%missing). There were no missing data for race/ethnicity, hypertension status, or diabetes status. This resulted in a final sample size of 510.

## Results

Table 1 depicts the characteristics of the study population. One hundred eighty-six participants (32.6%) had CAC present with scores ranging from 0 to 5098 Agatston (mean = 33, standard deviation = 385). Participants with CAC were older, male, white, had higher BMI, smoked, were diabetic, were hypertensive, had hyperlipidemia, and had a first degree relative with heart disease (p < 0.05 for all variables) compared to participants without calcification. The prevalence of CAC was similar in different education level groups, those reporting high or low depressive symptomatology, and those actively or passively responding to unfair treatment. Among those reporting any discrimination, 46.8% were African American, 36.1% were Hispanic, and 17.1% were white. Discrimination was reported by 35.5% of participants with coronary calcification and by 38.6% of participants without calcification (p = 0.50).

Results of the simple logistic regression model are shown in Table 2. Age and BMI were significantly associated with CAC. Men were 2.5 times more likely to have CAC present compared to females, while African Americans and Hispanics were 45% and 63%, respectively, less likely to have calcification compared to whites. Those who smoked, had diabetes, had hypertension, and had hyperlipidemia were more likely to have CAC present. Those with a 1^st ^degree relative with heart disease were approximately 1.5 times more likely to have CAC present. Education, depressive symptomatology, response to unfair treatment, and discrimination were not associated with CAC presence in these unadjusted models.

Race/ethnicity did not modify the relationship between discrimination and CAC. However, response to unfair treatment was found to significantly modify this relationship (p < 0.001). Hence, the simple logistic regression model assessing the relationship between discrimination and CAC and the multiple logistic regression results were stratified by active and passive response to unfair treatment. The sensitivity analysis found depression symptomatology to decrease the fit of the model; hence, it was not included in the final adjusted model.

The simple logistic regression models for discrimination and CAC stratified by response to unfair treatment are presented in Table 3. Among those who passively responded, participants who had experienced discrimination were 25% more likely to have CAC present, although this was not statistically significant. Table 4 provides results for the adjusted logistic regression model. Among those who actively responded to unfair treatment, only increasing age, being male, and being Hispanic were significantly associated with the presence of CAC. Among those who passively responded to unfair treatment, increasing age, being male, being African American, and having a positive smoking status were significantly associated with CAC. Interestingly, the odds of having CAC present were approximately 3 times higher for those who experienced discrimination and passively responded to unfair treatment.

## Discussion

This study of asymptomatic U.S. adults of different racial/ethnic identity is the first to our knowledge to support the association between experiencing racial discrimination and an increased risk of coronary artery calcification, a marker for atherosclerosis. Our results contradict two other studies that have investigated the influence of racial discrimination or unfair treatment and subclinical atherosclerosis[28,36]. Both previous studies were restricted to women and both found that "everyday" discrimination was associated with subclinical coronary artery disease, measured by coronary calcium [28] and intima-media thickness,[36] although one reported the association only among African American females and the relationship was not statistically significant[36]. However, when both studies restricted this association to racial discrimination, the association was no longer apparent. The authors concluded that it is not the attribution of discrimination but the experience of chronic discrimination that influences CAC. Neither study assessed how response to unfair treatment modified the association between self-reported discrimination and sub-clinical atherosclerosis. Our findings parallel studies that have found discrimination to be associated with hypertension among those who passively respond, or internalize their response, to unfair treatment[9,16,17]. Hence, it appears that coping mechanisms, such as speaking out in response to racist events, mitigates the impact of racial discrimination on CAC. These results remained significant after adjusting for smoking status and BMI and suggest future interventional studies are needed that empower individuals and communities to address and respond to everyday inequalities.

Several potential mechanisms linking psychosocial stressors such as self-reported discrimination to the development of coronary artery calcified plaque have been proposed[10]. Inflammatory induction is a pathophysiologic process that may be mediated by psychosocial stressors. Emerging evidence indicates that CVD development may involve the release of cytokines such as interleukin-6 and tumor necrosis factor α in an inflammatory response to epithelial damage stimulated by acute stressors[37]. Other possible mechanisms involve adverse health behaviors such as smoking, alcohol consumption, or poor diet in response to stress, which may contribute to risk,[38] although our results found several of these factors (i.e., smoking, and BMI) did not account for all of the adverse effect from racial discrimination.

The strengths of this study include the use of a validated instrument to measure discrimination and response to unfair treatment, the inclusion of multiracial/ethnic asymptomatic adults, and the use of the MSCT scan to detect coronary calcification. However, our results are subject to a number of limitations. We attempted to measure cumulative discrimination by determining whether participants *ever *experienced racial discrimination, although it is possible that recall may not be complete. In addition, we did not measure discrimination attributable to other characteristics, such as gender. The cross-sectional nature of the study precludes any statements about causal associations.

Previous research has confirmed that the experience of discrimination or unfair treatment may act as a stressor and that the appraisal of stress may also be important to measure[18,20]. Future investigations should include measures of discrimination attributed to multiple characteristics, whether psychosocial factors are intermediate factors in this association, and the moderating effects of coping resources. Potential variation by gender, race, and level of educational attainment should also be incorporated. Finally, the measure of discrimination investigated should reflect a lifecourse perspective and account for cumulative experiences of unfair treatment that may influence the disease process since atherosclerosis develops over an extended period. Moreover, factors that may impact how self-reported and perceived racism is reported should be examined. Variations on how one interprets discrimination, whether due to social status, geographic variation, or personal history, may affect how discrimination is measured[39]. For example, one study was able to extract thoughts or reports of past racist events among its participants only when they went into in-depth discussions[40]. Also, types of racist experiences in society have changed over time from more overt events to more subtle ones [41], such as suppression in social status and its impact on home ownership or higher educational opportunities.

## Conclusions

The persistence of racial disparities of CVD warrants investigations regarding the contribution of subjective experiences of discrimination and unfair treatment. Stress-related health literature has the potential to provide future direction. Focused efforts to elucidate whether racial and ethnic minorities differ in response to stressful situations, or types of stressful situations, may provide valuable insight for prevention and amelioration of CVD burden. Finally, because of the temporal lag between subclinical atherosclerosis and clinical coronary heart disease, the use of CAC as an endpoint should be emphasized in future epidemiologic studies of racial disparities in CVD.

## Abbreviations

BMI: Body mass index; CAC: Coronary artery calcification; CES-D: Center for Epidemiologic Studies Depression Scale; CHD: Coronary heart disease; CI: Confidence interval; CT: Computed tomography; CVD: Cardiovascular disease; EOD: Experiences of Discrimination instrument; mmHg: Millimeters of mercury; MSCT: Muli-slice computed tomography; NorTex: North Texas Primary Care Practice Based Research Network; NTHH: North Texas Healthy Heart study; ORs: Odds ratio

## Competing interests

The authors declare that they have no competing interests.

## Authors' contributions

RC conceived of the study, design and analyzed the data, and was the primary writer of the manuscript. KMC and KGF assisted in methodology of the study, assisted in data analyses, edited the manuscript. JC oversaw all labs analyses and methodology and edited the manuscript. AE oversaw daily study recruitment and execution of the project and edited the manuscript. CC, JV, RY, and DS all recruited study participants and were involved in manuscript development and editing. All authors read and approved the final manuscript.

## Pre-publication history

The pre-publication history for this paper can be accessed here:

http://www.biomedcentral.com/1471-2458/10/285/prepub
